# Microstructural White Matter Properties Mediate the Association between *APOE* and Perceptual Speed in Very Old Persons without Dementia

**DOI:** 10.1371/journal.pone.0134766

**Published:** 2015-08-07

**Authors:** Erika J. Laukka, Martin Lövdén, Grégoria Kalpouzos, Goran Papenberg, Lina Keller, Caroline Graff, Tie-Qiang Li, Laura Fratiglioni, Lars Bäckman

**Affiliations:** 1 Aging Research Center, Karolinska Institutet and Stockholm University, Stockholm, Sweden; 2 Division of Neurogeriatrics, Department of Neurobiology, Care Sciences and Society, Stockholm, Sweden; 3 Department of Geriatric Medicine, Karolinska University Hospital Huddinge, Stockholm, Sweden; 4 Department of Clinical Science, Intervention and Technology, Karolinska University Hospital Huddinge, Stockholm, Sweden; 5 Stockholm Gerontology Research Center, Stockholm, Sweden; University of Oxford, UNITED KINGDOM

## Abstract

**Background:**

Reduced white matter integrity, as indicated by lower fractional anisotropy (FA) and higher mean diffusivity (MD), has been related to poorer perceptual speed (PS) performance. As the ε4 allele has been associated with lower white matter integrity in old age, this represents a potential mechanism through which *APOE* may affect PS.

**Objective:**

To examine whether the association between *APOE* and PS is mediated by white matter microstructure in very old persons without dementia.

**Method:**

Participants were selected from the population-based SNAC-K study. After excluding persons with dementia, preclinical dementia, and other neurological disorders, 652 persons (age range 78–90) were included in the study, of which 89 had data on diffusion tensor imaging (DTI). We used structural equation modeling to form seven latent white matter factors (FA and MD) and one latent PS factor. Separate analyses were performed for FA and MD and mediational analyses were carried out for tracts where significant associations were observed to both *APOE* and PS.

**Results:**

*APOE* was associated with white matter microstructure in 2 out of 14 tracts; ε4 carriers had significantly lower FA in forceps major and higher MD in the cortico-spinal tract. Allowing the white matter microstructure indicators in these tracts to mediate the association between *APOE* and PS resulted in a markedly attenuated association between these variables. Bootstrapping statistics in the subsample with DTI data (*n* = 89) indicated that FA in forceps major significantly mediated the association between *APOE* and PS (indirect effect: -0.070, 95% bias corrected CIs -0.197 to -0.004).

**Conclusion:**

Lower white matter integrity may represent one of several mechanisms through which *APOE* affects PS performance in elderly persons free of dementia and preclinical dementia.

## Introduction

It is well documented that persons carrying the Apolipoprotein E (*APOE* ε4) allele have a higher risk of developing dementia compared to carriers of other allelic variants [1–2]. *APOE* has also been extensively studied in relation to cognitive functioning in normal aging. Although many studies report that cognitively healthy adults who carry the ε4 allele demonstrate poorer cognitive performance [3–4], the mechanisms underlying these associations are poorly understood. We showed that ε4 carriers performed at a lower level compared to ε4 non-carriers on tasks measuring episodic memory and perceptual speed (PS). After excluding persons who developed dementia within the next six years, only the association to PS remained significant [5]. Thus, the association between *APOE* and cognition may, at least in part, reflect dementia-related alterations in the brain. On the other hand, *APOE* was still associated with PS after excluding persons who later developed dementia. Thus, it may be the case that *APOE* influence cognition through pathways not directly linked to the dementia process. There is also the possibility of a shared mechanism that influences performance in both normal and pathological aging.

One factor that may contribute to the effect of *APOE* on PS in elderly persons without dementia is microstructural white matter integrity. Associations between white matter microstructure, as measured by diffusion tensor imaging (DTI), and PS have been documented in elderly persons. Specifically, reduced white matter integrity, indicated by lower fractional anisotropy (FA) or higher mean diffusivity (MD), has been related to poorer PS performance [6–8]. Using data from the Swedish National Study on Aging and Care in Kungsholmen (SNAC-K), we observed an association between white matter microstructure and PS in persons 78 years and older [9].

After reaching a peak in early adulthood, white matter integrity is negatively associated with increasing age [10–11]. However, there is substantial heterogeneity in indicators of white matter integrity in elderly samples that cannot be accounted for by age. Genetic factors represent one possible source of this heterogeneity [12–13], and *APOE* has been put forward as a potential candidate in this context. A main function of the APOE protein is the transport of lipids, which are important components of myelin. Carriers of the ε4 allele are thought to have reduced capacity for myelin repair [14–15], which may affect white matter integrity in old age. In line with this view, most studies on middle aged and older persons report that ε4 carriers have poorer white matter integrity compared to non-carriers [13]. However, results are not entirely consistent. For example, a recent study targeting persons between 21 and 70 years found increased radial diffusivity and MD in ε4 carriers, but failed to show allelic effects on FA [16]. Another study with persons 73 years of age, performed by Lyall et al. [17], showed deleterious effects of the ε4 allele on FA in right ventral cingulum and left inferior longitudinal fasciculus. Possible reasons for the observed discrepancies are differences in sample age and type of DTI measure [13].

The question whether the effect of *APOE* on cognition is mediated by white matter microstructure was recently addressed in a in a sample of 73-year-olds (the same sample as in Lyall et al. [17]). This study found that the effect of *APOE* on PS and general intelligence, but not on memory, was partially mediated by FA in one of the examined white matter tracts, left inferior longitudinal fasciculus [18]. However, more studies are needed in this area.

The purpose of the current study was to investigate the extent to which white matter microstructure (FA and MD) mediates the association between *APOE* and PS in very old persons without dementia. The analyses were performed in persons in a sample aged ≥78 years, where we had previously demonstrated an association between white matter microstructure and PS. A strength of this sample is the possibility to exclude persons with dementia as well as persons who develop dementia during the follow-up period (up to six years). Thus, we provide analyses of a very old sample, screened for both dementia and preclinical dementia, which represents a group that has rarely been examined in the context of *APOE* and white matter microstructure.

## Methods

### Participants

**Fig 1:**
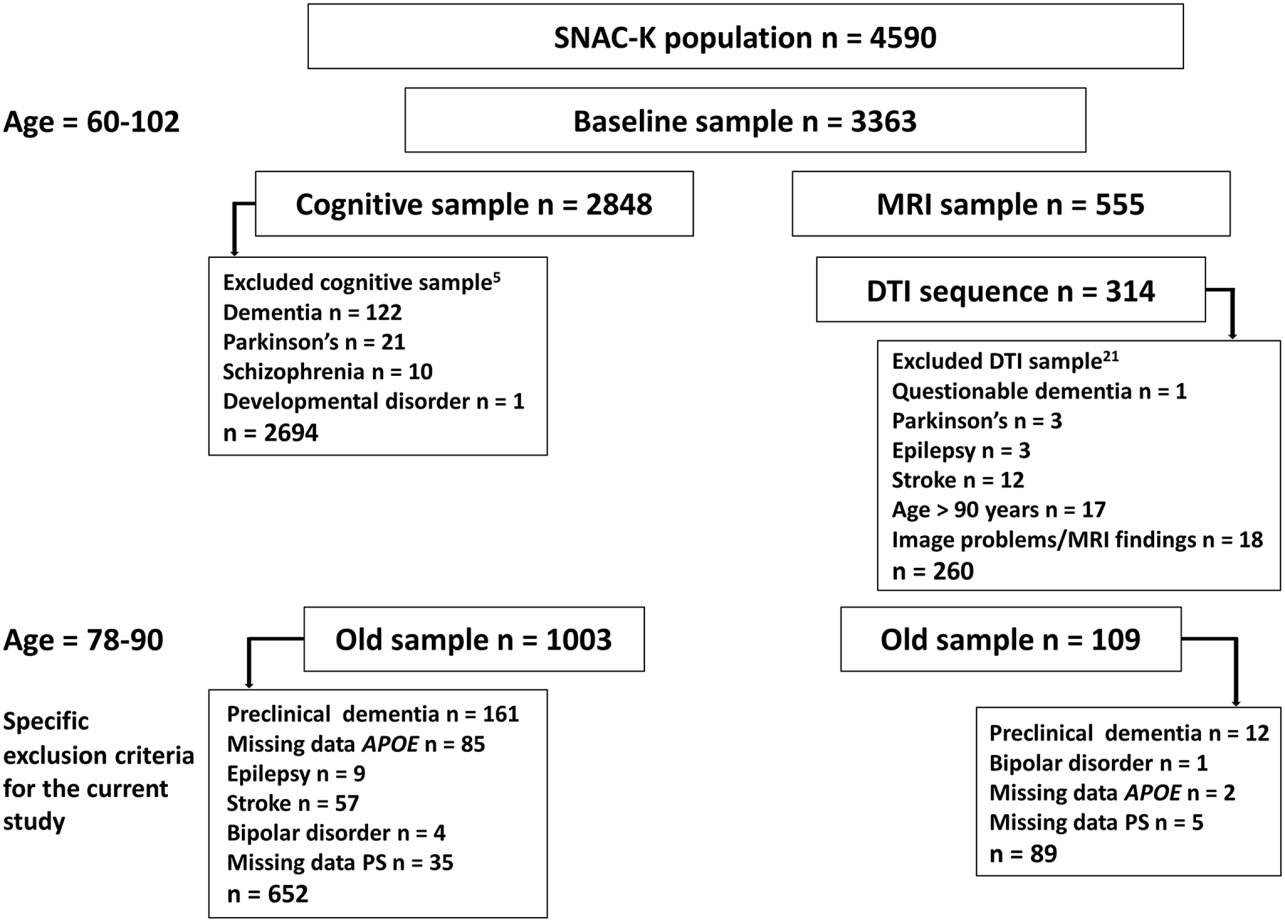
Number of participants and exclusion criteria for the total sample and the DTI subsample. SNAC-K = Swedish National study on Aging and Care in Kungsholmen, MRI = magnetic resonance imaging, DTI = diffusion tensor imaging, *APOE* = apolipoprotein E, PS = perceptual speed.

Participants were selected from the population-based SNAC-K study [19]. The original SNAC-K population consisted of 4590 alive and eligible persons who were ≥ 60 years old and lived on the island of Kungsholmen in central Stockholm, Sweden. These persons belonged to pre-specified age cohorts (60, 66, 72, 78, 81, 84, 87, 90, and 90+ years) and had been randomly selected based on birth dates retrieved from population records. Between 2001 and 2004, 3363 persons accepted to participate in the baseline examination consisting of a nurse interview and a medical examination. Due to additional refusals (*n* = 390), low Mini-Mental State Examination [20] scores (< 10, *n* = 106), death (*n* = 10), and other reasons (*n* = 9), the sample with data on the cognitive test battery consisted of 2848 persons [5]. The SNAC-K magnetic resonance imaging (MRI) sample (*n* = 555) was taken from non-institutionalized, non-disabled, and non-demented SNAC-K participants who were eligible for MRI assessment. Recruitment was done consecutively between September 2001 and October 2003 until the predefined sample size was reached. Of these, 314 persons were scanned with an identical DTI protocol, and thus constitute the DTI sample [21]. Average time between the neuropsychological testing and MRI assessment was 2 weeks. The samples that underwent cognitive testing [5] and DTI [21] in SNAC-K have been described in detail elsewhere. For the present study, we included persons who were between 78 and 90 years old, where we had previously demonstrated an association between white matter microstructure and PS, and who had data on *APOE* and PS. Persons with chronic diseases that might affect cognitive performance or white matter microstructure, such as Parkinson’s disease, epilepsy, stroke, schizophrenia, and bipolar disorder were excluded. Dementia was diagnosed according to DSM-IV criteria [22] in relation to the medical examination at each data collection wave. We excluded everyone with a dementia diagnosis at baseline or at either of the two follow-ups (after 3 and 6 years). After these exclusions, the effective sample consisted of 652 persons (mean age = 82.15, SD = 4.09, 67.94% female), of which 89 had DTI data. For an overview of the exclusion criteria for these two samples, the reader may consult Fig 1. Descriptive information for the subsample that only had cognitive data (*n* = 563) and the DTI subsample (*n* = 89) is presented in Table 1. Importantly, there were no differences between these samples with regard to *APOE* genotype. There were also no difference with regard to sex distribution and years of education. However, persons in the DTI subsample were positively selected in that they were younger, had higher cognitive performance, and fewer cardiovascular diseases (CVDs) than persons who only had cognitive data (Table 1). Selectivity of the DTI subsample was computed for age, Mini-Mental State Examination scores, and number of CVDs according to the formula (M_DTI sample_ − M_total sample_)/SD_total sample_. Analyses showed that selectivity based on these background variables was small (range = 0.18–0.22 SDs). Within the DTI subsample, *APOE* ε4 carriers were slightly better educated and had fewer vascular risk factors compared to ε4 non-carriers (Table 2), mainly due to a lower prevalence of hypertension (*p* < 0.05).

**Table 1:** Background information and data on perceptual speed for the cognitive and DTI subsamples.

	Cognitive sample (*n* = 563)	DTI sample (*n* = 89)	*P*
Age, *M* ± SD	82.27 ± 4.23	81.41 ± 3.01	0.02
Sex, *n* (%) female	385 (68.38)	58 (65.17)	0.55
Education, *M* ± SD	10.69 ± 3.84	11.48 ± 4.15	0.07
MMSE, *M* ± SD	28.55 ± 1.48	28.91 ± 1.07	0.01
*APOE*, *n* (%) any ε4	141 (25.04)	23 (25.84)	0.87
ε22, *n* (%)	1 (0.18)	0 (0.00)	
ε23, *n* (%)	61 (10.83)	10 (11.24)	
ε33, *n* (%)	355 (63.06)	56 (62.92)	
ε24, *n* (%)	16 (2.84)	2 (2.25)	
ε34, *n* (%)	115 (20.43)	20 (22.47)	
ε44, *n* (%)	10 (1.78)	1 (1.12)	
Aggregated cardiovascular risk factors, *M* ± SD	1.07 ± 0.75	1.04 ± 0.94	0.80
Diabetes, *n* (%)^a^	48 (8.63)	9 (10.23)	0.63
High cholesterol, *n* (%), ≥6.5 mmol/L	72 (12.97)	12 (13.64)	0.86
Hypertension stage 2, *n* (%)^b^	362 (64.30)	54 (60.67)	0.51
Obesity, *n* (%), ≥ 30 kg/m^2^	64 (11.62)	12 (13.48)	0.61
Current smoking, *n* (%)	57 (10.16)	6 (6.74)	0.31
Aggregated cardiovascular diseases, *M* ± SD	0.68 ± 0.90	0.46 ± 0.72	0.01
Atrial fibrillation, *n* (%)	138 (24.51)	10 (11.24)	0.01
Heart failure, *n* (%)	100 (17.76)	10 (11.24)	0.13
Ischemic heart disease, *n* (%)	142 (25.22)	21 (23.60)	0.74
PS			
Digit cancellation, *M* ± SD	15.62 ± 3.58	16.56 ± 3.44	0.02
Pattern comparison, *M* ± SD	11.41 ± 3.02	12.74 ± 2.81	< 0.01

*Note*. DTI = diffusion tensor imaging, MMSE = Mini-Mental State Examination, *APOE* = apolipoprotein E, PS = perceptual speed.

^a^ based on self-report, inpatient register, use of oral glucose-lowering agent or insulin injection, or HbA1c ≥5.4%

^b^ ≥160/100 mm Hg or use of antihypertensive agents

**Table 2:** Background information for the DTI subsample (*n* = 89) stratified by *APOE* ε4 genotype.

	No ε4 (*n* = 66)	Any ε4 (*n* = 23)	*p*
Age, *M* ± SD	81.66± 3.10	80.69 ± 2.69	0.18
Sex, *n* (%) female	44 (66.67)	14 (60.87)	0.62
Education, *M* ± SD	10.96 ± 3.89	12.96 ± 4.60	0.05
MMSE, *M* ± SD	28.98 ± 0.98	28.70 ± 1.29	0.27
Aggregated cardiovascular risk factors, *M* ± SD	1.17 ± 0.99	0.70 ± 0.70	0.04
Aggregated cardiovascular diseases, *M* ± SD	0.41 ± 0.63	0.61 ± 0.94	0.26

*Note*. DTI = diffusion tensor imaging, *APOE* = apolipoprotein E, MMSE = Mini-Mental State Examination.

Written informed consent was obtained from all participants. The SNAC-K project has been approved by the ethical committee at Karolinska Institutet (dnr 01–114), Stockholm, Sweden, and the research was conducted according to the ethical guidelines from the Declaration of Helsinki.

### Genotyping

DNA was extracted from peripheral blood samples using standard methods. Thereafter, genotyping of *APOE* (rs7412, rs429358) was performed using MALDI-TOF analysis on the Sequenom MassARRAY platform at the Mutation Analysis Facility, Karolinska Institutet. In brief, DNA was amplified using primer pairs designed to amplify the desired single-nucleotide polymorphism (SNP) loci. A single base extension reaction with a primer adjacent to the SNP site was then performed with mass modified dideoxynucleotides. The resulting allele-specific extension products were differentiated by their respective mass, which were analyzed by MassARRAY MALDI-TOF mass spectrometry. Quality control was performed at the DNA-sample level, assay level, and the level of multiplex assay pool. Successful genotyping call rate for *APOE* was over 99% and genotyping results were in Hardy Weinberg equilibrium (rs7412: *p* = 0.34; rs429358: *p* = 0.18). Allelic frequencies (see Table 1) were in accordance with reports from other European samples. In the present study, *APOE* was dichotomized into any ε4 (one or two) versus no ε4.

### MRI acquisition and preprocessing of DTI images

The MRI measurements were conducted using a 1.5 Tesla scanner (Philips Intera, Netherlands). DTI data were acquired using a single-shot diffusion-weighted echoplanar imaging sequence with the following parameters: FOV = 230 × 138 mm^2^; 128 x 77 matrix; TE = 104 ms; TR = 6838 ms; slice thickness = 5 mm with 1 mm gap; b-value 600 s/mm^2^. A DTI scheme with 6 non-collinear diffusion-weighting gradient directions was used to determine the diffusion tensor set.

A detailed description of how the DTI data were preprocessed and how FA and MD were derived has been provided elsewhere [21]. In short, after diffusion tensor calculation, these parameters were derived on a voxel-by-voxel basis using three steps: (1) estimation of eigenvalues and eigenvectors of the diffusion tensor using the single-value decomposition algorithm; (2) calculation of MD as the mean of the diagonal elements; and (3) calculation of FA according to its definition [23]. The FA data were further processed using tract-based spatial statistics (TBSS [24]) in FSL [25]. Here, the mean FA image was thinned to create a mean FA skeleton, which represents the centerlines of all tracts common to the sample. The mean skeleton was thresholded and binarized at FA > 0.2 to reduce the likelihood of partial voluming. Individual skeleton images were then derived by projecting each participant’s aligned FA data onto this skeleton. Finally, the MD images were processed based on the results of the processing of the FA images.

**Fig 2:**
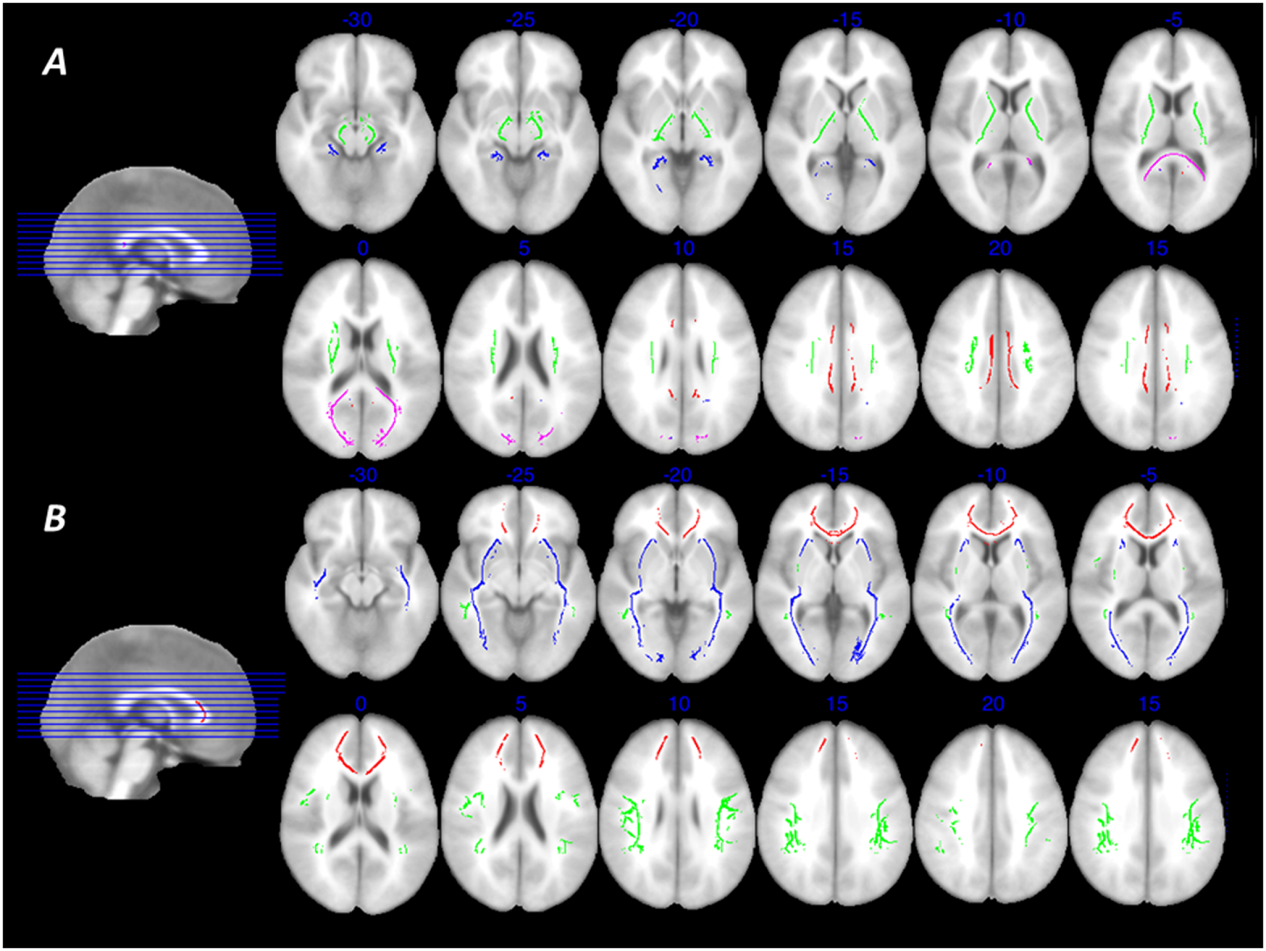
Examined white matter tracts. Regions of interests in the TBSS skeleton from which mean fractional anisotropy and mean diffusivity were extracted for each individual. The regions of interest were based on modified probabilistic template masks emanating from the Catani and JHU white-matter tractography atlases (see Methods section for details). ***A***: Red, cingulum cingulate gyrus; blue, cingulum hippocampus; green, corticospinal tract; violet, forceps major. ***B***: Red, forceps minor; blue, inferior fronto-occipital fasciculus; green, superior longitudinal fasciculus. The backdrop image is the MNI ICBM template.

We produced masks of seven tracts of interest in each hemisphere, using the procedures validated by Lövdén et al. [21]: the cingulate gyrus part of cingulum (CCG), the portion of cingulum that extends to the hippocampus (CHC), the corticospinal tract (CS), the forceps major (FMAJ), the forceps minor (FMIN), the inferior fronto-occipital fasciculus (IFOF), and the superior longitudinal fasciculus (SLF). These 14 masks (7 tracts x 2 hemispheres) were used to extract mean FA and MD data from each individual’s skeleton image (Fig 2). All masks were based on the JHU white-matter tractography atlas [26–27], except the CS mask where the Catani tractography atlas [28–29] fitted the skeleton better. The size of the masks was generally similar across hemispheres, as were the mean FA and MD values [21]. The tract-based approach was chosen because we had previously shown that this model fits the data well, whereas a model with a general white matter factor does not give an adequate representation of individual differences in this dataset [21].

### Cognitive testing

For a full description of the testing procedures, the reader may consult Laukka et al. [5]. PS was measured by means of two paper-and-pencil tests. For digit cancellation [30], participants were presented with eleven rows of random digits ranging from 1 to 9. They were instructed to sequentially go through the rows as quickly as possible and cross out every “4” they encountered. The outcome for this task was number of correctly crossed 4s within 30 sec. For pattern comparison [31], participants were presented with 30 pairs of line-segment patterns. The instruction was to go through the patterns as quickly as possible and mark them as “same” or “different”. The outcome for this task was mean number of correct classifications across two trials, each lasting 30 sec.

### Cardiovascular risk factors and white matter hyperintensities

Two aggregated sum scores were created for cardiovascular risk factors (CRFs) and CVDs (Tables 1 and 2). Information for these aggregated scores was collected through self-report, clinical examination, medication lists, laboratory data, and the computerized Stockholm inpatient register. For a detailed description of the assessment of these variables, see Welmer et al. [32].

White matter hyperintensities (WMH) were manually drawn on FLAIR images by a single rater and further interpolated on the corresponding T1 images to compensate for the gap between slices in FLAIR. We adjusted for intracranial volume (ICV) using an analysis of covariance approach: adjusted WMH = raw WMH volume − b x (ICV − mean ICV), where b is the slope of regression of WMH volume on ICV. This procedure is often used when analyzing volumetric data because a simple ratio adjustment approach tends to yield positively skewed values [33–34]. Data on white matter microstructure and WMH for the DTI subsample are presented in Table 3.

**Table 3:** Data on white matter microstructure and WMH for the DTI subsample (*n* = 89).

	*M*	*SD*
FA (left+right)		
CCG	38.86	2.41
CHC	38.54	2.16
CS	55.35	2.16
FMAJ	56.18	2.83
FMIN	49.58	3.09
IFOF	45.26	2.19
SLF	40.85	2.52
MD (left+right)		
CCG	85.15	3.96
CHC	105.59	7.61
CS	76.63	2.82
FMAJ	81.52	5.52
FMIN	85.09	5.26
IFOF	87.27	4.57
SLF	80.09	4.59
Global WMH (mL)	10.79	13.07

*Note*. WMH = white matter hyperintensities adjusted for ICV, DTI = diffusion tensor imaging, FA = fractional anisotropy, MD = mean diffusivity, CCG = cingulum cingulate gyrus, CHC = cingulum hippocampus, CS = corticospinal tract, FMAJ = forceps major, FMIN = forceps minor, IFOF = inferior fronto-occipital fasciculus, SLF = superior longitudinal fasciculus. The unit for MD was 10^−9^ m^2^/s. All FA and MD variables have been multiplied with 100 to make the variances more similar to those of the cognitive variables.

### Statistical analyses

Differences between persons with and without DTI data and with and without an *APOE* ε4 allele were examined by *χ* ^2^ (categorical variables) and *t* tests (continuous variables). All other analyses were performed with AMOS (IBM SPSS 22). Variables analyzed with structural equation modeling (SEM) displayed acceptable skewness and kurtosis, indicating normally distributed variables [35], except for the ICV-adjusted WMH volume which displayed normal distribution after log-transformation.

**Fig 3:**
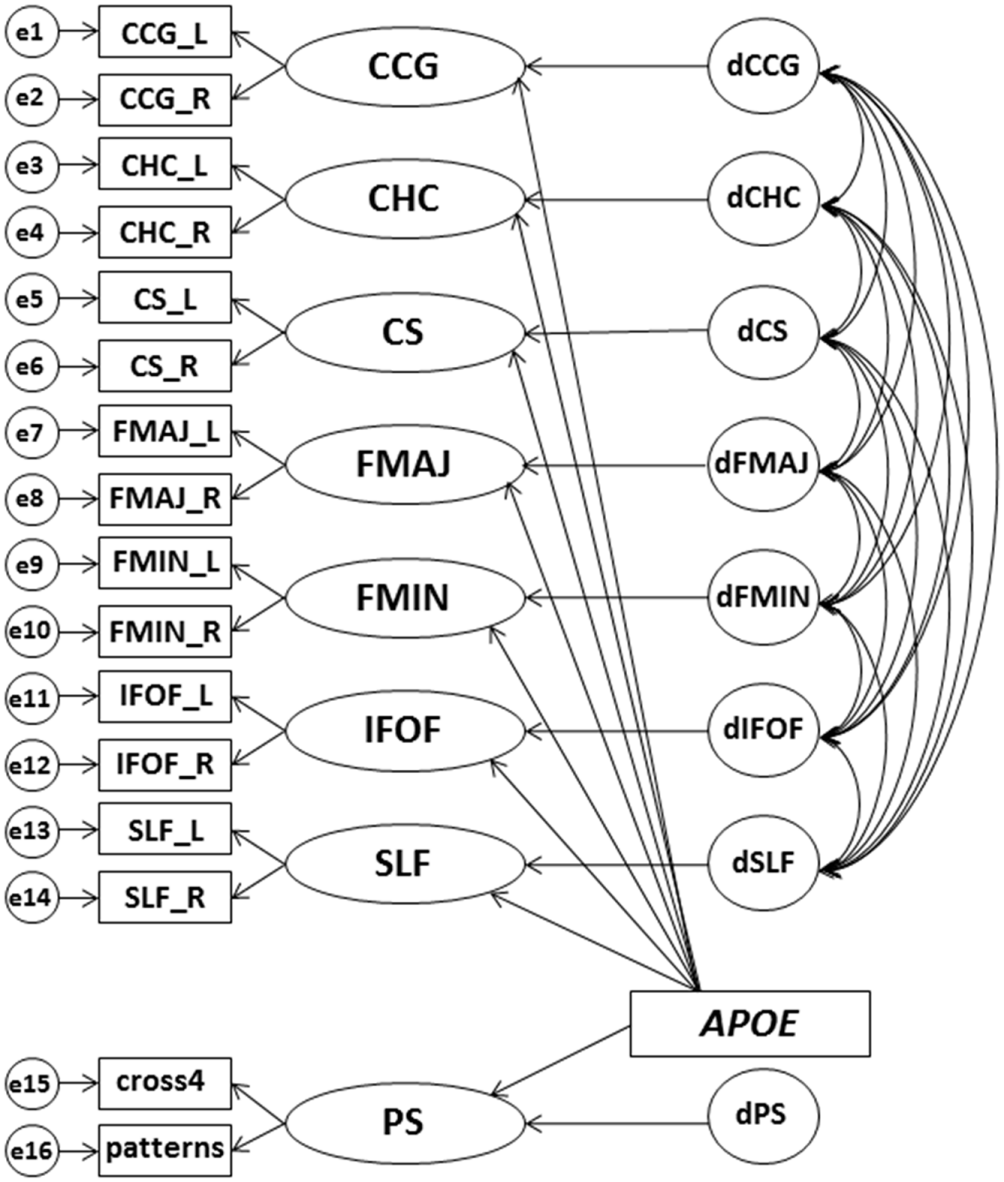
Structural equation model for the effect of *APOE* on the seven latent white matter factors and the latent PS factor. Latent factors are depicted with circles, observed variables with rectangles, regressions with one-headed arrows, and covariances with two-headed arrows. CCG = cingulum cingulate gyrus, CHC = cingulum hippocampus, CS = corticospinal tract, FMAJ = forceps major, FMIN = forceps minor, IFOF = inferior fronto-occipital fasciculus, SLF = superior longitudinal fasciculus, PS = perceptual speed. Separate models were fit for fractional anisotropy and mean diffusivity. All analyses were controlled for age (not included in figure).

We created separate SEM models for FA and MD, where the latent white matter factors represent individual differences common across the left and right hemisphere for each tract (Fig 3). Model fit was evaluated with the Comparative Fit Index (CFI) and the Root-Mean-Square Error of Approximation (RMSEA). Acceptable model fit was defined as a CFI above 0.95 and an RMSEA below 0.08 [35]. For the analyses in the DTI subsample, some models included negative error variances, likely due to small sample size [36]. These error variances were fixed to 0.

Chronological age was included as an additional regressor on all latent factors in the model. Thus, age was covaried in all analyses. For the cognitive data, the latent PS factor represents the common variance for the two PS measures. Standardized loadings on the latent factors and correlations among the latent white matter factors are available as supporting information (S1–S3 Tables).

First, we determined the associations between the latent tract factors and *APOE*, and second, we examined whether the effect of *APOE* on PS was statistically mediated by white matter microstructure. This was only done for those tracts that showed reliable associations to both *APOE* and PS [37]. We calculated the effect size, expressed as *r* ^2^, of all associations and % change in effect size after mediation for the *APOE*-PS association ((*r* ^2^ after mediation–*r* ^2^ before mediation)/*r* ^2^ before mediation). Bootstrapping of the indirect effect of *APOE* on PS was performed to confirm the robustness of the results. Resampling with replacement was performed 5000 times in the DTI subsample, which only included persons with complete data. The threshold for statistical significance was set to *p* < 0.05.

### Handling missing values

All models with missing data were estimated with Full Information Maximum Likelihood (FIML) [38–39]. This state-of-the art method uses all available information for estimating parameters that involve missing values, making it possible to include persons with missing DTI data without performing any data imputation. This method is preferable to other procedures used to deal with missing values (e.g., list wise deletion, mean imputation), because it generates less biased population estimates [39]. Analyses using list-wise deletion restrict the sample to a select number of persons, making estimates from this sample select in relation to the population, whereas the FIML approach corrects for this selectivity when estimating population values. Another advantage of using FIML is that the latent PS factor is estimated with more precision when the full sample is included. Therefore, the models were estimated using all available data on the cognitive measures (*n* = 652) and on the DTI measures (*n* = 89), as previously done by Lövdén et al. [40].

Although we believe that the estimates obtained with FIML are more precise and less biased than estimates from models restricted to the sample with DTI data, we chose to also report the results from the analyses using list-wise deletion. This was done to demonstrate that the same pattern of results was obtained with both methods, despite the relatively large amount of missing values for the DTI data. Thus, we repeated all analyses in the sample with complete data on cognition and DTI. Results from both the total sample (*n* = 652) and the DTI subsample (*n* = 89) are reported throughout the paper, except for the bootstrapping analyses (see previous section), where only the DTI subsample was used.

## Results

**Fig 4:**
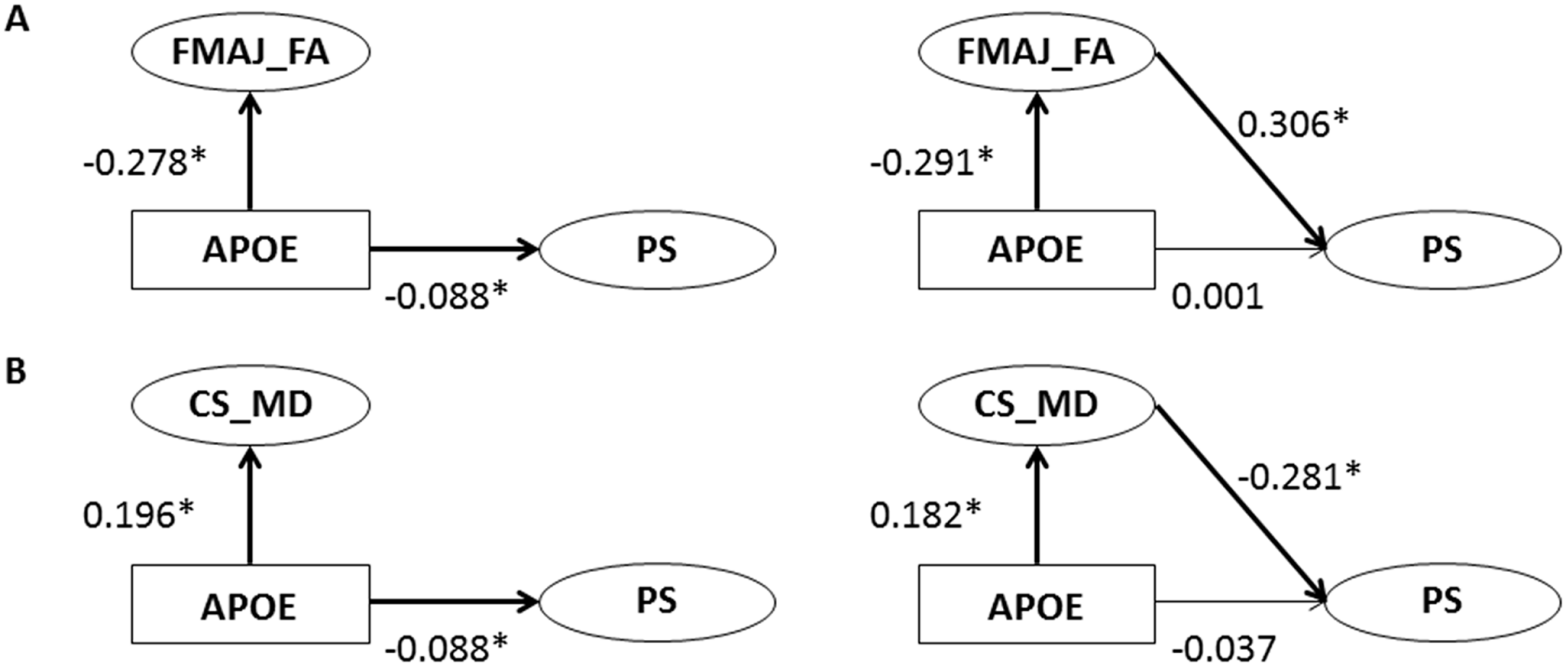
Associations between *APOE* and PS, before and after entering white matter microstructure as a mediator in the total sample (*n* = 652). Analyses were performed in separate models for FMAJ FA (A) and CS MD (B). Latent factors are depicted with circles, observed variables with rectangles, and regressions with one-headed arrows. FA = fractional anisotropy, MD = mean diffusivity, FMAJ = forceps major, CS = corticospinal tract, PS = perceptual speed. All analyses were controlled for age (not included in figure). * *p* < 0.05.

We obtained excellent fit for a model combining seven latent white matter tracts, one latent PS factor, and *APOE* (manifest variable) regressed on the latent factors (Fig 4) for FA (total sample: *χ* ^*2*^ = 96.68, df = 99, *n* = 652, CFI = 1.00, RMSEA = 0.00; DTI subsample: *χ* ^*2*^ = 96.10, df = 100, *n* = 89, CFI = 1.00, RMSEA = 0.00). For MD, we obtained acceptable model fit (total sample: *χ* ^*2*^ = 163.87, df = 99, *n* = 652, CFI = 0.96, RMSEA = 0.03; DTI subsample: *χ* ^*2*^ = 162.64, df = 100, *n* = 89, CFI = 0.95, RMSEA = 0.08). Table 4 shows the associations between *APOE* and the latent white matter factors for the DTI subsample. These associations were virtually identical when estimated in the total sample (data not shown). In general, ε4 carriers had lower FA and higher MD values compared to non-carriers. However, significant associations were only observed between *APOE* and FA in FMAJ and between *APOE* and MD in CS.

**Table 4:** Associations between *APOE* (any ε4 vs. no ε4) and white matter microstructure in seven tracts (*n* = 89).

	FA		MD	
	Standardized estimate	*p*	Standardized estimate	*p*
CCG	-0.11	0.33	0.17	0.13
CHC	0.09	0.57	-0.02	0.87
CS	0.00	0.99	0.23	0.03
FMAJ	-0.33	< 0.01	0.13	0.24
FMIN	-0.13	0.24	0.17	0.11
IFOF	-0.14	0.21	0.12	0.29
SLF	-0.08	0.47	0.19	0.07

*Note*. *APOE* = apolipoprotein E, FA = fractional anisotropy, MD = mean diffusivity, CCG = cingulum cingulate gyrus, CHC = cingulum hippocampus, CS = corticospinal tract, FMAJ = forceps major, FMIN = forceps minor, IFOF = inferior fronto-occipital fasciculus, SLF = superior longitudinal fasciculus. All effects were adjusted for age.

Second, we performed mediational analyses, including those tracts that were significantly related to *APOE*. Note that these tracts were also related to PS, as has previously been reported [9]. The first model included *APOE*, FA in FMAJ, and PS, and showed good fit (total sample: *χ* ^*2*^ = 8.98, df = 6, *n* = 652, CFI = 0.99, RMSEA = 0.03; DTI subsample: *χ* ^*2*^ = 9.29, df = 8, *n* = 89, CFI = 0.99, RMSEA = 0.04). Next, we compared the strength of the association between *APOE* and PS in a model with no mediation to a model where FA in FMAJ was allowed to mediate the effect between *APOE* and PS (Fig 4A). The second model (Fig 4B), including *APOE*, MD in CS, and PS, also showed good fit (total sample: *χ* ^*2*^ = 6.50, df = 6, *n* = 652, CFI = 1.00, RMSEA = 0.01; DTI subsample: *χ* ^*2*^ = 6.79, df = 7, *n* = 89, CFI = 1.00, RMSEA = 0.00). The results of the mediational analyses indicate that the influence of *APOE* on PS is substantially reduced when FA in FMAJ serves as a mediator (Table 5). Similarly, allowing MD in CS to mediate the association between *APOE* and PS resulted in a reduced and non-significant association between *APOE* and PS. The mediator-associated reductions of the effect of *APOE* on PS were 100% for FA in FMAJ and 82% for MD in CS, with a somewhat attenuated mediation effect in the DTI subsample (70 and 54%, respectively; Table 5). Note, however, that the pattern was very similar for both samples, and that the mediation effect was present also in the smaller DTI subsample.

**Table 5:** Mediational models for *APOE*, white matter microstructure, and PS in the total sample (*n* = 652) and in the DTI subsample (*n* = 89).

	Total sample							DTI subsample						
	Before mediation			After mediation				Before mediation			After mediation			
	STD estimate	*p*	*r* ^2^	STD estimate	*p*	*r* ^2^	% change	STD estimate	*p*	*r* ^2^	STD estimate	*p*	*r* ^2^	% change
FA FMAJ														
*APOE* → PS	-0.088	0.041	0.0077	0.001	0.988	0.000	-100%	-0.154	0.137	0.0237	-0.084	0.432	0.0071	-70%
*APOE* → FMAJ	-0.278	0.002	0.0773	-0.291	0.001	0.0847		-0.305	0.002	0.0930	-0.305	0.002	0.0930	
FMAJ → PS	-	-	-	0.306	0.030	0.0936		-	-	-	0.231	0.037	0.0534	
MD CS														
*APOE* → PS	-0.088	0.041	0.0077	-0.037	0.490	0.0014	-82%	-0.154	0.137	0.0237	-0.104	0.317	0.0108	-54%
*APOE* → CS	0.196	0.037	0.0384	0.182	0.045	0.0331		0.212	0.040	0.0449	0.203	0.047	0.0412	
CS → PS	-	-	-	-0.281	0.034	0.0790		-	-	-	-0.250	0.027	0.0625	

*Note*. *APOE* = apolipoprotein E, PS = perceptual speed, DTI = diffusion tensor imaging, STD = standardized, FA = fractional anisotropy, MD = mean diffusivity, FMAJ = forceps major, CS = corticospinal tract. All effects were adjusted for age.

To further confirm the robustness of the results, bootstrapping of the indirect effect of *APOE* on PS was performed in the DTI subsample (*n* = 89), again using age as a covariate. The standardized indirect effect of *APOE* on PS via FA in FMAJ was -0.070, with a 95% bias corrected confidence interval (CI) of -0.197 to -0.004. The CIs did not include 0 for this model, thus verifying the robustness of the mediational effect. For the second model, the standardized indirect effect of *APOE* on PS via MD in CS was -0.051, 95% CI -0.170 to 0.001. Thus, the results from the second model should be interpreted with caution, as it was not confirmed to be stable by the bootstrapping analyses (*p* = 0.06). Moreover, the effect of *APOE* on MD in CS would not survive a Bonferroni correction, if applied to these data. Adjusting the α-level according to the number of examined tracts would render a new α-level of 0.05/14 = 0.004. If applying this adjusted α-level, only the effect of *APOE* on FA in FMAJ would remain significant.

As complementary analyses, we performed the same mediation models described above, adding additional covariates. The effects of these additional covariates were very similar in the total sample and the DTI subsample. Controlling for sex, education, CRFs, CVDs, and WMH volume in addition to age did not affect the results for FA, whereas for MD the mediation effect was reduced (Total sample: 39% change in effect size vs. 82%; DTI sample: 28% change vs. 54%). The largest effect originated from controlling for WMH volume, rendering a 61% change in effect size as compared to 82% (DTI sample: 32% vs. 54%). A slight reduction of the mediation effect for MD in CS was also observed when controlling for vascular disease burden, resulting in a 75% change in effect size as compared to 82% (DTI sample: 49% vs. 54%).

## Discussion

The results from this study render support to the hypothesis that white matter microstructure partly mediate the effect of *APOE* on PS in very old persons without dementia. First, we examined the associations between *APOE* and the white matter tract factors. The general pattern showed deleterious effects of the ε4 allele on white matter microstructure, however, significant associations were only observed for FMAJ (FA) and CS (MD). This observation is in line with previous studies, where *APOE* ε4 has often been associated with lower white matter integrity [13], [16–17]. Note, however, that the effect of *APOE* was small and only significant for two out of fourteen tracts. Applying a Bonferroni correction to these data would result in a significant effect of *APOE* for only one tract (FA in FMAJ). There have also been previous reports of lack of effects of *APOE* on white matter integrity in older participants, especially in studies using FA as the indicator variable [16], [41–42]. FA and MD capture different aspects of white matter integrity. Animal studies suggest that axial and radial diffusivity, on which both FA and MD are based, are sensitive to different aspects of white matter changes, where axial diffusivity may be more sensitive to axonal differences [43] and radial diffusivity more sensitive to myelin changes [44]. Thus, depending on the pattern of white matter changes, FA and MD may be more or less correlated [45–46] and it is possible that genetic factors influence white matter differently for FA and MD, as well as for different tracts.

White matter microstructure was also related to PS (see also Laukka et al. [9]). White matter tracts are essential for efficient transfer of information between different brain regions. Thus, it is not surprising that several studies have shown white matter microstructure to be associated with PS [6–8]. Disconnection of cortical areas and functional disruption of neurocognitive networks, as a result of white matter deterioration, have been proposed to be one mechanism behind age-related deficits in cognitive functioning [47–48]. Thus, factors contributing to the intactness of white matter may help preserve cognitive performance, in particular PS, in old age. FMAJ is a large white matter fiber tract connecting the occipital lobes through the corpus callosum. Similarly, inferior longitudinal fasciculus, which was found to significantly mediate the effect of *APOE* on PS in the study by Lyall et al. [18], connects the occipital and temporal lobes. It is biologically plausible that less efficient communication flow in these tracts leads to slower information processing speed. That said, the association between white matter microstructure and PS should be interpreted with some caution, given the possibility that a sensorimotor component played a role in this association. Most PS tasks, including the ones used in this study, are dependent on motor abilities. The CS, which was the second tract found to mediate the *APOE*-PS association in the present study, transfers information from the motor cortex to the spinal cord and is therefore essential for sensorimotor functions used for speeded paper-and-pencil tasks [49]. Thus, observed links between white matter microstructure, in particular in CS, and PS should be viewed with this in mind.

Allowing white matter microstructure to mediate the association between *APOE* and PS resulted in markedly reduced, and non-significant, effects of *APOE* on PS. Although other mechanisms are conceivable, this observation is consistent with the view that the *APOE* polymorphism affects PS performance partly through its differential effects on white matter microstructure. A possible mechanism for this influence is through its role as transporter of lipids such as cholesterol—essential for myelin production, maintenance, and repair [50]. On the one hand, accelerated rates of age-related myelin breakdown due to impaired repair capacity may occur in ε4 carriers [14,51]. However, *APOE* has been associated with white matter microstructure also in early adulthood [41], and even among infants [52]. Thus, *APOE* ε4 may contribute to lower levels of myelination early in life, accelerated rate of myelin breakdown in late life, or both.

The mediational effect observed here was strongest for FA in FMAJ, and this effect was also confirmed to be robust by bootstrapping analyses. The mediational effect for MD in CS was somewhat smaller and the indirect effect of *APOE* on PS only reached marginal significance in the bootstrapping analyses. Moreover, the effect of *APOE* on MD in CS would not survive a Bonferroni correction. Mediational analyses were not carried out for tracts that were not significantly related to *APOE*, although there was a tendency for a negative influence of the ε4 genotype for most white matter tracts.

Persons carrying the ε4 allele have a higher risk of developing dementia [1–2]. Although we excluded persons with dementia and even preclinical dementia, it is still not possible to conclude that the participants were free of dementia-related pathology. Dementia-related brain changes may be present years to decades prior to diagnosis [53–55], which makes it difficult to completely exclude persons in a pre-dementia phase even when follow-up data is available. Thus, we cannot rule out the possibility that the effect of *APOE* was, at least in part, due to dementia-related brain changes. However, the important functions of the APOE protein in the brain, and the findings that *APOE* may affect white matter microstructure also at younger ages [41,52], make it unlikely that the influence of *APOE* on white matter solely reflects premorbid pathology among ε4 carriers.

Studies involving DTI generally apply a threshold when assessing indicators of white matter integrity, and thus only include normal-appearing white matter. However, it may still be necessary to control for WMH in studies of white matter microstructure [8,56]. WMH do not always have distinct boundaries [57], which makes it difficult to separate between normal tissue and tissue affected by a proximate lesion. Furthermore, *APOE* is associated with degree of WMH burden [58]. Toward this end, we found that the mediational effect for MD was weakened when controlling for WMH load. This suggests that macrostructural damage may play a role in these associations, perhaps especially so in a very old sample where WMH are more abundant.

A major strength of the present study is that we had access to follow-up information on most subjects, and thus were able to exclude persons with incipient dementia. The presence of persons in a pre-dementia phase may otherwise be a confounding factor in many studies on *APOE*. We demonstrated the same mediation pattern in a larger, more representative sample, and in a subsample with DTI data. Further, both white matter integrity and PS were examined at the latent level, thus increasing reliability of measurements. Although three measures per latent factor would be preferable, we only had access to two measures per factor. Another limitation is that the DTI measurements were not of modern quality; large and anisotropic voxels may have introduced partial volume effects (grey/white mixture) that could not be completely accounted for by TBSS processing. Note also that the analyses were based on cross-sectional data. Longitudinal studies are needed to deepen our understanding of the influence of *APOE* on age-related changes in brain integrity and cognitive functioning. Future studies should also investigate the effect of *APOE* on white matter microstructure in more specific genotype groups, which was not possible in the present study due to sample-size restrictions.

The goal of any statistical analysis is to estimate population values with the least possible bias. In this respect, list-wise deletion has repeatedly been shown to fare considerably worse than FIML estimation. This is because the remaining sample (in this case, the subsample with DTI data), after deleting cases with missing values, is select in relation to the population [39]. The FIML approach corrects for this selectivity when estimating population values. Note that the FIML approach does not impute any missing values, but takes into account all available information in the model to recover the estimates. However, the FIML approach only recovers the true estimates if the missing-at-random (MAR) assumption [59] holds. MAR assumes that any systematic associations between patterns of missingness and the actual missing scores can be statistically accounted for by the data present in the model. In our case, the models include several predictors of missingness (e.g., cognitive data, age), so that any remaining association between missingness and the outcome variables is likely to be negligible. Note also that selectivity was relatively small already from the outset. Even if some remaining violation of MAR would be present, it has been convincingly shown that, in many realistic scenarios, an erroneous assumption of MAR (e.g., failing to take into account a cause or correlate of missingness) has a small impact on estimates and standard errors [60]. This stands in contrast to the major impact the list-wise deletion procedure may have on the estimates [39]. Although low coverage (i.e., large amount of missing values for the DTI data) may lead to estimation problems, we noted no such problems in the present analysis. In fact, the FIML approach was helpful for estimating the cognitive part of the models. Thus, we argue that it is advantageous to use FIML in the context of large-scale neuroimaging studies when this is possible. In cases where the MAR assumption is reasonably well met, this method helps alleviating the problem of positively selected neuroimaging samples relative to full study samples.

To conclude, the current results suggest that lower white matter integrity represents one possible mechanism through which *APOE* affects PS performance in elderly persons without dementia. The effect of *APOE* on PS is not large. However, there is evidence that *APOE*-related cognitive deficits may be exacerbated in combination with other vulnerability factors, such as head injury [61], vascular disease [62], and accumulation of *β*-amyloid [63], thereby exerting large effects for some individuals. Thus, *APOE*, and the mechanisms through which its allelic variants affect cognition are important to consider in relation to brain aging.

## Supporting Information

S1 Table Standardized loadings on the latent factors in the structural equation models for fractional anisotropy (FA) and mean diffusivity (MD) in the total sample (*n* = 652) and the DTI subsample (*n* = 89). (DOCX)

S2 Table Correlations among the latent white matter tract factors in the structural equation models for fractional anisotropy (below the diagonal) and mean diffusivity (above the diagonal) in the total sample (*n* = 652). (DOCX)

S3 Table Correlations among the latent white matter tract factors in the structural equation models for fractional anisotropy (below the diagonal) and mean diffusivity (above the diagonal) in the DTI subsample (*n* = 89). (DOCX)
