# Supplementary material for: Microstructural White Matter Properties Mediate the Association between APOE and Perceptual Speed in Very Old Persons without Dementia
Source: PLoS One. 2015 Aug 7;10(8):e0134766. doi: 10.1371/journal.pone.0134766 (PMC4529164; doi:10.1371/journal.pone.0134766)
Supplement: S1 Table — (DOCX) [file pone.0134766.s001.docx]

**Microstructural white matter properties mediate the association between *APOE* and perceptual speed in very old persons without dementia**

**PLOSOne**

Erika J. Laukka*, Martin Lövdén, Grégoria Kalpouzos, Goran Papenberg, Lina Keller, Caroline Graff, Tie-Qiang Li, Laura Fratiglioni, Lars Bäckman

*Corresponding author. E-mail: Erika.Jonsson.Laukka@ki.se

**S1 Table. Standardized loadings on the latent factors in the structural equation models for fractional anisotropy (FA) and mean diffusivity (MD) in the total sample (*n* = 652) and the DTI subsample (*n* = 89).**

|  | Total sample | | DTI subsample | |
| --- | --- | --- | --- | --- |
|  | FA | MD | FA | MD |
| CCG_L | 0.87 | 0.82 | 0.86 | 0.79 |
| CCG_R | 0.85 | 0.86 | 0.85 | 0.84 |
| CHC_L | 0.53 | 0.69 | 0.53 | 0.68 |
| CHC_R | 0.61 | 0.84 | 0.60 | 0.84 |
| CS_L | 0.95 | 0.97 | 0.95 | 0.96 |
| CS_R | 0.92 | 0.93 | 0.92 | 0.92 |
| FMAJ_L | 0.94 | 0.85 | 0.94 | 0.83 |
| FMAJ_R | 0.83 | 0.87 | 0.82 | 0.85 |
| FMIN_L | 0.97 | 0.92 | 0.97 | 0.91 |
| FMIN_R | 0.94 | 0.91 | 0.94 | 0.90 |
| IFOF_L | 0.95 | 0.91 | 0.95 | 0.90 |
| IFOF_R | 0.88 | 0.91 | 0.88 | 0.90 |
| SLF_L | 0.92 | 0.94 | 0.92 | 0.94 |
| SLF_R | 0.88 | 0.91 | 0.88 | 0.91 |
| PS |  |  |  |  |
| Digit cancellation | 0.72 | 0.72 | 1.00 | 1.00 |
| Pattern comparison | 0.78 | 0.78 | 0.56 | 0.56 |

*Note.* DTI = diffusion tensor imaging, CCG = cingulum cingulate gyrus, CHC = cingulum hippocampus, CS = corticospinal tract, FMAJ = forceps major, FMIN = forceps minor, IFOF = inferior fronto-occipital fasciculus, SLF = superior longitudinal fasciculus, PS = perceptual speed.
