# Supplementary material for: Microstructural White Matter Properties Mediate the Association between APOE and Perceptual Speed in Very Old Persons without Dementia
Source: PLoS One. 2015 Aug 7;10(8):e0134766. doi: 10.1371/journal.pone.0134766 (PMC4529164; doi:10.1371/journal.pone.0134766)
Supplement: S2 Table — (DOCX) [file pone.0134766.s002.docx]

**Microstructural white matter properties mediate the association between *APOE* and perceptual speed in very old persons without dementia**

**PLOSOne**

Erika J. Laukka*, Martin Lövdén, Grégoria Kalpouzos, Goran Papenberg, Lina Keller, Caroline Graff, Tie-Qiang Li, Laura Fratiglioni, Lars Bäckman

*Corresponding author. E-mail: Erika.Jonsson.Laukka@ki.se

**S2 Table. Correlations among the latent white matter tract factors in the structural equation models for fractional anisotropy (below the diagonal) and mean diffusivity (above the diagonal) in the total sample (*n* = 652).**

| Factor | CCG | CHC | CS | FMAJ | FMIN | IFOF | SLF |
| --- | --- | --- | --- | --- | --- | --- | --- |
| CCG | - | 0.33* | 0.58* | 0.60* | 0.74* | 0.63* | 0.70* |
| CHC | 0.54* | - | 0.37* | 0.73* | 0.34* | 0.59* | 0.29* |
| CS | 0.77* | 0.55* | - | 0.68* | 0.67* | 0.80* | 0.84* |
| FMAJ | 0.74* | 0.36* | 0.55* | - | 0.59* | 0.99* | 0.71* |
| FMIN | 0.94* | 0.46* | 0.69* | 0.66* | - | 0.73* | 0.75* |
| IFOF | 0.75* | 0.72* | 0.60* | 0.68* | 0.76* | - | 0.86* |
| SLF | 0.89* | 0.69* | 0.82* | 0.57* | 0.77* | 0.76* | - |

CCG = cingulum cingulate gyrus, CHC = cingulum hippocampus, CS = corticospinal tract, FMAJ = forceps major, FMIN = forceps minor, IFOF = inferior fronto-occipital fasciculus, SLF = superior longitudinal fasciculus.

* *p* < 0.05
